# Concurrent hyperthyroidism and papillary thyroid cancer: a fortuitous and ambiguous case report from a resource-poor setting

**DOI:** 10.1186/s13104-016-2178-0

**Published:** 2016-07-26

**Authors:** Benjamin Momo Kadia, Christian Akem Dimala, Ndemazie Nkafu Bechem, Desmond Aroke

**Affiliations:** 1 Presbyterian General Hospital Acha-Tugi, Acha-Tugi, North West Region Cameroon; 2 Faculty of Epidemiology and Population Health, London School of Hygiene and Tropical Medicine, London, UK; 3 Health and Human Development (2HD) Research Group, Douala, Cameroon; 4 Penka Michel District Hospital, Penka-Michel, Cameroon; 5 Banso Baptist Hospital, Kumbo, North West Region Cameroon

**Keywords:** Thyroid cancer, Hyperthyroidism, Papillary thyroid cancer

## Abstract

**Background:**

Concurrent thyroid cancer (TC) and hyperthyroidism (HT) is rare though increasingly being reported. HT due to TC is much rarer and more challenging especially in Africa where TC and HT have significant case fatality rates.

**Case presentation:**

We present a 37-year-old Cameroonian female who had been on irregular regimens of propranolol and digoxin as treatment for worsening palpitations for 12 months. She came to our district hospital for her propranolol medication refill. We fortuitously identified features of HT and found a left uninodular goiter with no cervical lymphadenopathy. She was referred for thyroid assessment which suggested primary HT and an enlarged heterogeneous left lobe with a well-defined homogenous solid mass. We restarted her on propranolol and referred her for a course of methimazole. At the referral hospital, she also underwent a left thyroid lobectomy. The resected lobe was sent for histopathology which revealed a neoplastic nodule with features suggestive of a papillary thyroid cancer (PTC) causing HT. The patient’s clinical progress postoperatively was good and there was regression of hyperthyroid symptoms.

**Conclusions:**

The historical, clinical, and laboratory findings were suggestive of HT due to PTC. A high index of suspicion, prompt referral and counter-referral lead to a positive outcome of such a rare case in a resource poor setting. We advocate for systematic and careful evaluation of all thyroid nodules.

## Background

Thyroid cancer (TC) is the most common malignant endocrine tumour worldwide [1–3] but accounts for only 1 % of global malignancies [4]. It is, thus, a relatively uncommon cancer [4]. Papillary thyroid cancer (PTC) is the predominant variant of TC [1, 3, 5, 6].

Hyperthyroidism (HT) is a state of excessive thyroid hormone production [1]. Its most common aetiologies are Graves’ disease, toxic uninodular goiter (usually a toxic adenoma) and toxic multinodular goiter (Plummer’s disease) [1].

The co-existence of TC and HT is rare, although there are increasing reports [7–11]. The association of TC and HT poses serious diagnostic, therapeutic and prognostic enigmas [8, 9, 12, 13]. More so, it is much rarer and more challenging for HT to be due to TC which is instead usually associated with euthyroidism [7–9, 13, 14].

In Africa, thyroid diseases carry significant morbidity and mortality with the leading causes being TC and HT [2]. This is accounted for by the late presentation of patients, lack of robust diagnostic facilities, and poor accessibility to healthcare resources [2].

We report here an additional and nearly missed case of concurrent PTC and HT from a remote district of Cameroon. We attributed the HT to the PTC.

## Case presentation

A 37-year-old woman purposely came to the outpatient department of our district hospital in the North West Region of Cameroon for a refill of her propranolol medication. She reported taking the drug routinely for palpitations. On further inquiry, she reported being well until 12 months prior to presentation when she suddenly became aware of her heartbeat, initially on moderate exertion and then even at rest. It had been recurrent and lasting several minutes each time it occurred. It progressively became associated with dyspnoea initially on moderate exertion and then on mild exertion, such that she had to quit farming which was her main source of income. After consulting at various health facilities in the North West Region of Cameroon, she was prescribed irregular and alternate daily regimens of 40 mg propranolol and 0.25 mg digoxin which she had been taking for 12 months at the time she presented at our hospital. Although the medications conferred her some relieve, she noticed a progressive weight loss over the 12-months period prior to consulting at our hospital, from 85 to 58 kg despite an abnormal increase in her appetite for food. She also experienced frequent watery stools (averagely six times daily). About 6 months prior to presentation, she complained of heat intolerance and profuse sweating resulting in sleeping with minimal clothing. In addition, she noticed spontaneous resting tremors of her hands which started at the same time as the heat intolerance. Three months prior to presentation at our hospital, she noticed a painless lump on her neck that progressively increased in size.

On reviewing her medical records, we noticed a number of investigations requested at various hospitals which she visited during the 8 months preceding her presentation at our hospital. These included electrocardiographs, echocardiograms, HIV tests, full blood counts, fasting blood sugar, thyroid hormone assays, which were all without particularity but for the electrocardiography results which always showed a sinus tachycardia. She has no known history of exposure to radiations or family history of malignancies.

**Fig. 1:**
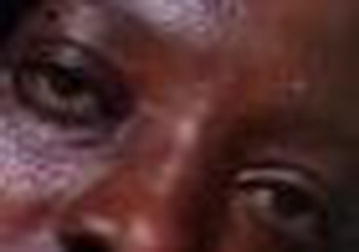
Eyes of the patient

**Fig. 2:**
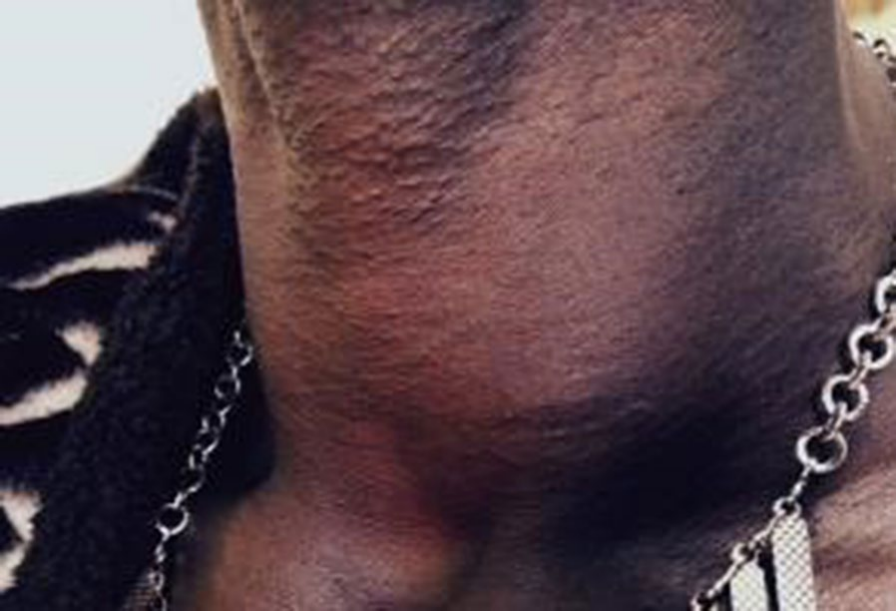
Anterolateral neck mass

Physical examination revealed a chronically ill-looking middle aged woman. Her eyes were normal (Fig. 1). Her voice was clear. She had fine resting tremors of her hands with her arms outstretched. There was a left anterolateral neck mass measuring 4 × 3 cm (Fig. 2). The mass was rubbery, mobile, non-tender, moved with swallowing, and not fixed to overlying or underlying tissue. No bruit was heard over the mass. There was no palpable cervical lymphadenopathy. Her vital signs were normal but for regular respiratory and pulse rates of 35 breaths/min and 104 beats/min respectively. Her BMI was 20.1 kg/m^2^. There was discrete bilateral pedal pitting oedema. The rest of the physical examination was without notable findings. In view of this presentation, a presumptive diagnosis of HT was made and we placed her on propranolol, 40 mg twice daily. Our hospital was not equipped with the necessary diagnostic tools, so we referred the patient to a regional hospital which is about 10 km from our locality. To confirm our diagnosis, we requested for a functional [serum T3, T4, Thyroid stimulating hormone (TSH)] and structural (ultrasound) assessment of the thyroid. The patient was counter-referred to us with the following results:

1. Functional assessment of the thyroid: levels of serum T3, T4, and TSH (Table 1).

**Table 1:** Functional assessment of thyroid gland of the patient

Test	Results	Normal range	Comment
T3	>5.0	(0.8–2.0) ng/mL	Elevated
T4	23.3	(5.0–13.0) ɱg/dL	Elevated
TSH	0.13	(0.4–7.0) ɱIU/mL	Low
2. Structural assessment of the thyroid (ultrasound scan): The left thyroid lobe appeared enlarged, heterogeneous, with a fairly iso-echoic, well-defined homogenous solid mass (3.6 × 1.8 × 2.9 cm in size). The right lobe was without particularity. No cervical lymphadenopathy was observed.

**Fig. 3:**
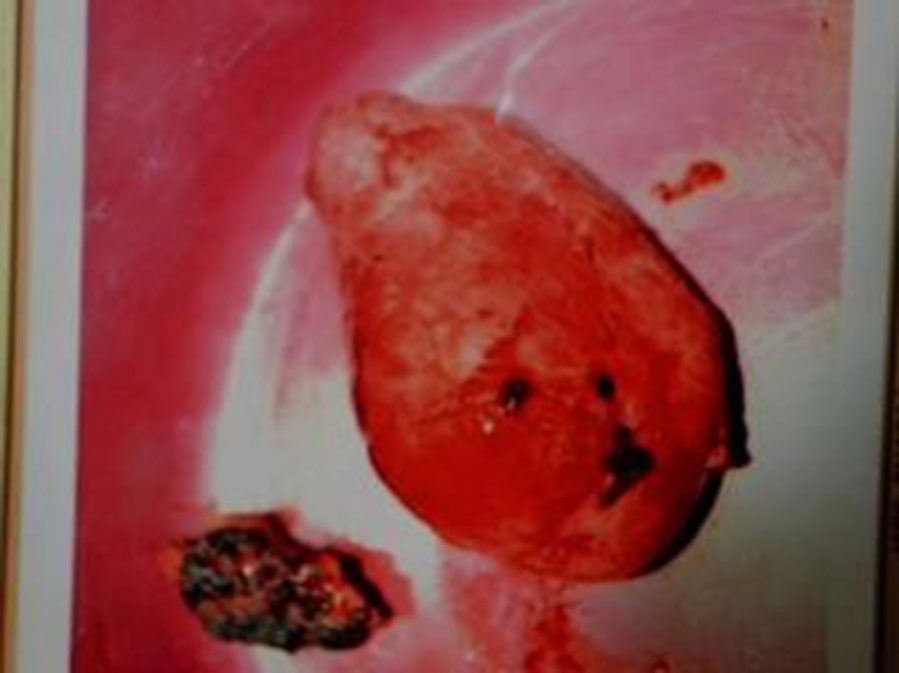
Resected thyroid lobe (2 fragments)

In view of these findings, we concluded on a primary HT most likely due to a toxic uninodular goiter. Again, due to the limited resources in our hospital, we referred the patient to a hospital which is over 30 km from our locality for initiation of a course of methimazole. She was placed on 60 mg methimazole daily, 4 weeks after which she underwent a left thyroid lobectomy. The resected lobe (Fig. 3) was sent for histopathology. The postoperative course was uneventful.

The lobe weighed 33.5 g and was received in formalin as two grey-tan soft tissue fragments: a large (6.5 × 5 × 3.5 cm) and a small (3 × 1.5 × 1 cm) fragment. Cutting demonstrated a red tan cut surface with a 3 × 2.3 × 2 cm nodule inside the bigger piece. Microscopic examination of representative sections of the large fragment with a full thickness section of the nodular structure revealed a nodular proliferation of enlarged pale cells with marginated chromatin and overlapping nuclei. Pink “bubble gum”-like colloid was focally noted. The lesion was partially encapsulated and displayed areas with fibrosis and more follicular appearance of the aggregates. Based on these, a histological diagnosis of PTC (pT2N0M0) was made.

**Fig. 4:**
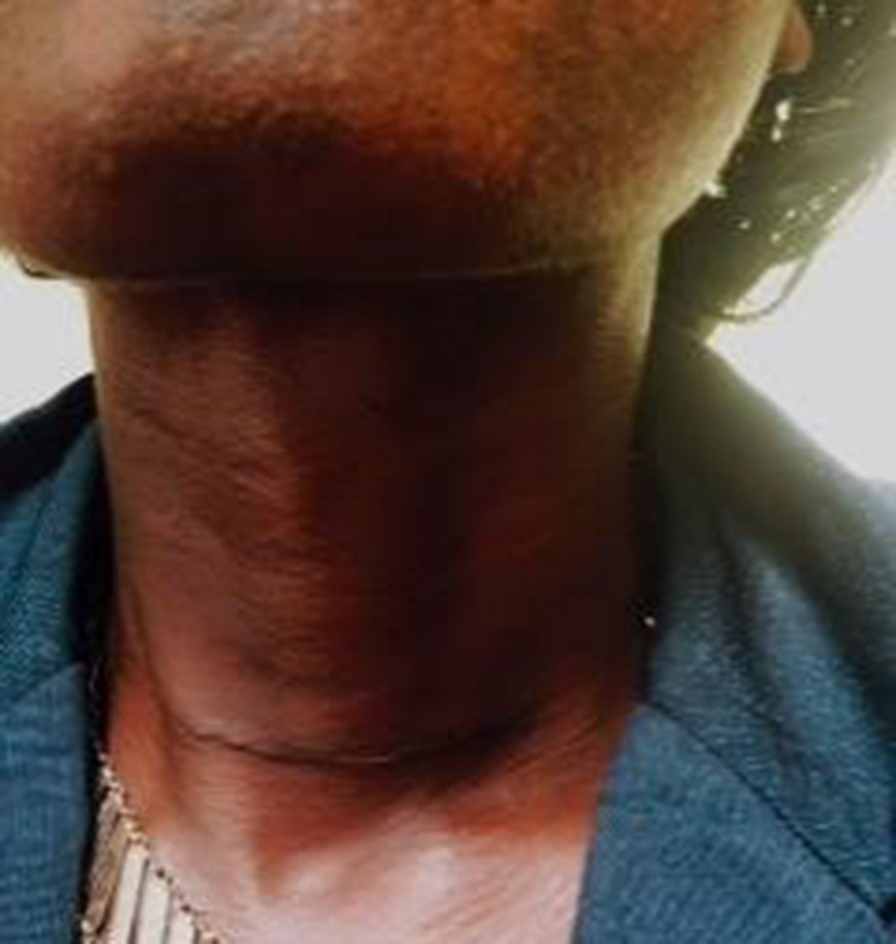
Incision site 2 weeks after lobectomy

We monitored the patient through scheduled regular visits and referrals. Figure 4 shows the Incision site on the anterior aspect of the neck 2 weeks after lobectomy. We observed a progressive decline in hyperthyroid symptoms and signs: the palpitations regressed as well as the diarrhoea and polyphagia; the heat intolerance regressed and 1 month postoperatively, her weight increased from 58 to 68 kg.

## Discussion

We report a fortuitous finding of concurrent HT and PTC from a resource-limited setting. The case is more perplexing as the HT seemed to stem from the PTC. It is a challenge to manage such a rare case from a remote district of Cameroon as the country has a poorly developed healthcare referral system and only 20 % of the population has access to referral centres [15].

Graves’ disease, though the most common cause of HT [1, 2, 16], is less likely the diagnosis since the goiter was not diffuse, the eyes were normal and there was no pretibial myxoedema. A hyperfunctioning thyroid nodule therefore took precedence as the probable aetiological diagnosis, with a toxic adenoma being the most likely aetiology. Since the patient’s TSH levels were low, the most appropriate next step would have been a thyroid scan (scintigraphy) to determine if the nodule is hot [1, 7, 17]. That notwithstanding, even if the HT were due to a hyperfunctioning (hot) nodule, it is very rare for such a nodule to have malignant potentials, thus, they are seldom biopsied after scintigraphy [7, 8, 17]. Being deficient in thyroid scan in our hospital facilities, the next means to confirm the characteristics of the thyroid nodule was by histopathological analysis. However, a limitation in the management of this case is that though histopathology can be done on a resected lobe, histopathology through fine needle aspiration cytology would have allowed for proper planning of the surgical approach to be used [7, 11, 17]. This is because it is suggested that toxic thyroid cancers are best treated by total thyroidectomy while incidental carcinomas can be managed by subtotal thyroidectomy or lobectomy [9].

Interestingly, histopathology revealed a PTC. Given the partial encapsulation of the neoplastic nodule in this case, the lesion could be further classified as an encapsulated variant of PTC. The differential diagnosis also included follicular adenoma with papillary hyperplasia but the extensive nuclear changes observed were typical of PTC (pT2N0M0). This case is again unusual in that most malignant thyroid tumours associated with HT that have been reported so far are papillary thyroid microcarcinomas (nodule <1 cm or pT1) [7]. Nonetheless, Mirfakhraee et al. in 2013 reported that amongst patients with hyperfunctioning TC, a greater rate of frank biochemical HT was observed in patients with larger nodules as in our case [8]. The many follicular cells observed in the neoplastic lesion were suggestive of follicular hyperactivity which is seen in toxic TC [7].

If HT is due to a tumour cell mass, demonstrated in most cases by clinical-histopathological correlation, a toxic TC is diagnosed [9]. An ambiguity, however, remains because a scintigraphically hot nodule was not confirmed. Concomitant TC and HT can either be in the form of a fortuitous malignancy in the thyroid gland of a clinically hyperthyroid patient or as a TC presenting with HT with the latter being rarer [9, 14, 17]. The two can be distinguished via histopathology, with the lack of hyperplastic thyroid tissue suggesting a hyperfunctioning TC [9] as in the case presented. Again, with retrospect to the lack of ultrasound findings of increase vascularity and diffuse enlargement of thyroid tissue, further credence is lent to the autonomous nature of the tumour cell mass [9]. In terms of risk factors, based on the study by Mirfakhraee et al., patients with malignant hot nodules seem more likely to be younger and females [8] as is the case with the patient presented.

Hyperthyroidism due to TC is explained by somatic mutations in TSH receptor genes of the cancer cells. These mutations lead to constitutive activation of intracellular cyclic Adenosine Monophosphate (cAMP) cascade which induces hormonogenesis and thus HT [7, 9].

## Conclusions

The historical, clinical, and laboratory findings of the case we report concurred with HT due to PTC. A high index of suspicion should be the attitude towards every thyroid nodule. Despite the difficult diagnostic and therapeutic terrain in our resource-limited setting, a good referral and counter-referral system lead to a positive outcome. We advocate for systematic and careful evaluation of all thyroid nodules.

## Abbreviations

HT: hyperthyroidism
PTC: papillary thyroid cancer
TC: thyroid cancer
TSH: thyroid stimulating hormone
pT2N0M0: tumour node metastasis of the TNM grading system
cAMP: cyclic Adenosine Monophosphate
